# ﻿A new species and a key to the genus *Leiurus* Ehrenberg, 1828 (Scorpiones, Buthidae) from Saudi Arabia

**DOI:** 10.3897/zookeys.1178.109083

**Published:** 2023-09-07

**Authors:** Abdulmani H. Al-Qahtni, Abdullah M. Al-Salem, Fahad Mesfer, Manal S. Al Balawi, Wasayf S. Allahyani, Abdulaziz R. Alqahtani, Ahmed Badry

**Affiliations:** 1 Department of Invertebrates, National Center for Wildlife, Saudi Arabia National Center for Wildlife Riyadh Saudi Arabia; 2 Department of Biology, College of Science, University of Bisha, P.O. Box 551, Bisha 61922, Saudi Arabia University of Bisha Bisha Saudi Arabia; 3 Department of Zoology, Faculty of Science, Al-Azhar University, Nasr City, Cairo, Egypt Al-Azhar University Cairo Egypt

**Keywords:** Description, identification key, Majami al-Hadb Protected Area, molecular phylogeny scorpion, taxonomy

## Abstract

A new species, *Leiurushadb* Al-Qahtni, Al-Salem, Alqahtani & Badry, **sp. nov.**, is described and illustrated from the Majami al-Hadb Protected Area in the Riyadh Province of Saudi Arabia. The new species is compared with species of *Leiurus* distributed in Saudi Arabia, especially *L.arabicus* Lowe, Yağmur & Kovařík, 2014. The integrated results indicate that the population found in Majami al-Hadb represents a distinct species, which is described herein. Moreover, the molecular analysis is conducted on the mitochondrial gene 16S rRNA to compare *L.hadb* sp. nov. with samples of *L.arabicus* and *L.haenggii* from Saudi Arabia. The analysis revealed a genetic divergence ranging from 6.0 to 12%. The combination of molecular evidence and morphological characteristics provides adequate support for recognizing the Majami al-Hadb population as a distinct species. Additionally, an identification key for the genus *Leiurus* found in Saudi Arabia is also provided.

## ﻿Introduction

The genus *Leiurus* was first introduced by Ehrenberg in Hemprich and Ehrenberg (1829) as a subgenus of *Androctonus*. While Vachon (1949) considered *L.quinquestriatus* the only species of the genus, several new species have been described in recent years, spanning a vast geographical area and a variety of habitats (Lourenço et al. 2002, 2006, 2018; Yağmur et al. 2009; Lowe et al. 2014; Lourenço and Rossi 2016; Lourenço 2019, 2020a, 2020b; Kovařík and Lowe 2020; Lourenço and El-Hennawy 2021). Recently, a new species of *Leiurus* was described by Lourenço (2022) from the Al-Anbâr Province in Iraq, bringing the total number of known species in this genus to 20. This increase in the number of *Leiurus* species was predicted in several previous publications as more specimens from distinct populations became available (Lourenço et al. 2002, 2006), which was confirmed particularly for the populations of the Middle East (Lowe et al. 2014; Lourenço 2022). In Saudi Arabia, there are four species of the genus *Leiurus*: *L.arabicus* Lowe, Yağmur & Kovařík, 2014 from the central Najd plateau to the east of Saudi Arabia, *L.brachycentrus* (Ehrenberg, 1829) known from Tihamah coastal plain in Saudi Arabia and Yemen, *L.haenggii* Lowe, Yağmur & Kovařík, 2014 from the coastal mountains of the Red Sea in Saudi Arabia, Yemen, and Oman, and *L.jordanensis* Lourenço, Modry & Amr, 2002, from sandstone cliffs isolated by dunes in Jordan and northern Saudi Arabia (Lowe et al. 2014; Alqahtani et al. 2019). Although significant efforts have been invested in the last years, some populations remain poorly defined, such as the one distributed in southwestern Saudi Arabia. Lowe et al. (2014) suggested that there might be other distinct populations that could be different from the populations of *L.arabicus* and *L.haenggii* based on morphological investigations.

In this study, we describe a new species of the genus *Leiurus* based on several specimens of the Majami al-Hadb Protected Area under an integrative taxonomic perspective, using morphological and molecular evidence.

## ﻿Materials and methods

A total of 11 specimens of *Leiurushadb* sp. nov. was collected from Majami al-Hadb Protected Area in the Riyadh Province between 23 and 25 May 2023, using ultraviolet light at night. The specimens were preserved in 96% alcohol and photographed using a Canon EOS 6D Mark II. The photographs were edited using Adobe Photoshop software. The measurements were taken in mm on preserved specimens, according to Stahnke (1970). Trichobothrial patterns followed (Vachon 1974), while morphological terminology mostly adhered to the conventions of Vachon (1952) and Hjelle (1990). Type material is deposited in the Museum of National Center for Wildlife, Riyadh (**NCWM**).

### ﻿Molecular analysis

The genomic DNA was isolated from five scorpion specimens of *L.hadb* sp. nov., using Qiagen DNA extraction kits following the manufacturer’s instructions. The amplified 16S rRNA gene products were purified and sequenced using invertebrate universal primers, as determined, and sequenced on an ABI 3500 automated sequencer (Applied Biosystems Inc., USA) and following Gantenbein et al. (1999). The sequences were edited using BioEdit v. 7.2.5 Hall (1999). The new sequences, generated from all five samples, were added to the sequences previously generated from four samples of *L.arabicus* and *L.haenggii* from Saudi Arabia by (Alqahtani and Badry 2020a). Also, additional sequences of species of *Leiurus* from Egypt, Oman, and Turkey, were retrieved from GenBank. The sequence of *Androctonuscrassicauda* (Olivier, 1807; AY156570.1) was downloaded as the outgroup. The sequences were aligned using the default settings of ClustalW in Mega 11 (Tamura et al. 2021), and nucleotide composition was calculated from the ingroup sequences only. Genetic distances (p-distances) for the entire data set were calculated using Mega 11 (Tamura et al. 2021). Phylogenetic analyses of the 16S data set (*N* = 18) were performed following Alqahtani and Badry (2020b). Maximum-parsimony and neighbor-joining analyses were conducted with PAUP* v. 4 (Swofford 2000) using heuristic clustering based on TBR branch swapping. A character was considered missing when a gap was present in an alignment. To assess the degree of confidence within the nodes, 1000 bootstrapping replicates and random additions of taxa were used (Felsenstein 2002). The best-fit nucleotide evolution models were preferred using MrModeltest v. 2.3 (Nylander 2004) based on the Akaike Information Criterion (Akaike 1973) in PAUP* v. 4 (Swofford 2000). To infer the geographic structure, Bayesian inference (BI) was implemented using MrBayes v. 3.1.2 (Ronquist et al. 2012) for a million generations, and output parameters were plotted with Tracer v. 1.7 (Rambaut et al. 2018).

## ﻿Taxonomic treatment

**Family Buthidae C.L. Koch, 1837**

**Genus *Leiurus* Ehrenberg, 1828**

### ﻿ Leiurus hadb

Taxon classificationAnimaliaScorpionesButhidae

Al-Qahtni, Al-Salem, Alqahtani & Badry sp. nov.

33BC364E-0A96-580B-9CFC-BCF643457756

https://zoobank.org/F662BF83-F4D4-4E6B-AD83-327FAEA735F0

**Figures 1.**
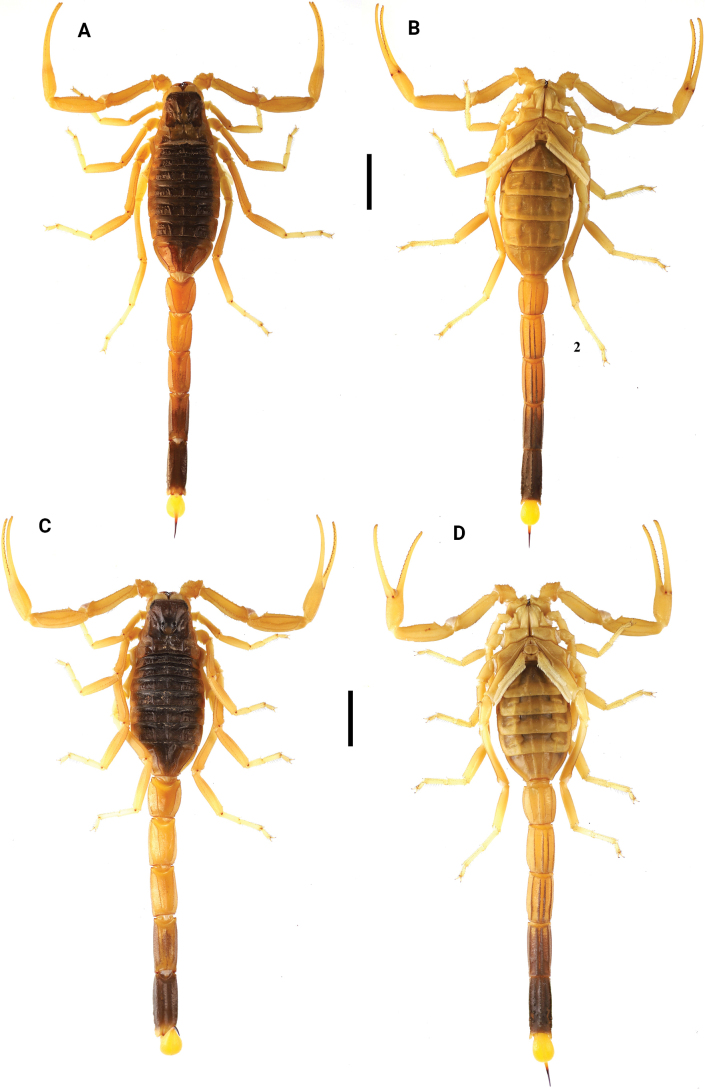
Habitus of *Leiurushadb* sp. nov., male holotype and female paratype **A** male in dorsal view **B** male in ventral view **C** female in dorsal view **D** female in ventral view. Scale bar: 10 mm.

**Figures 2.**
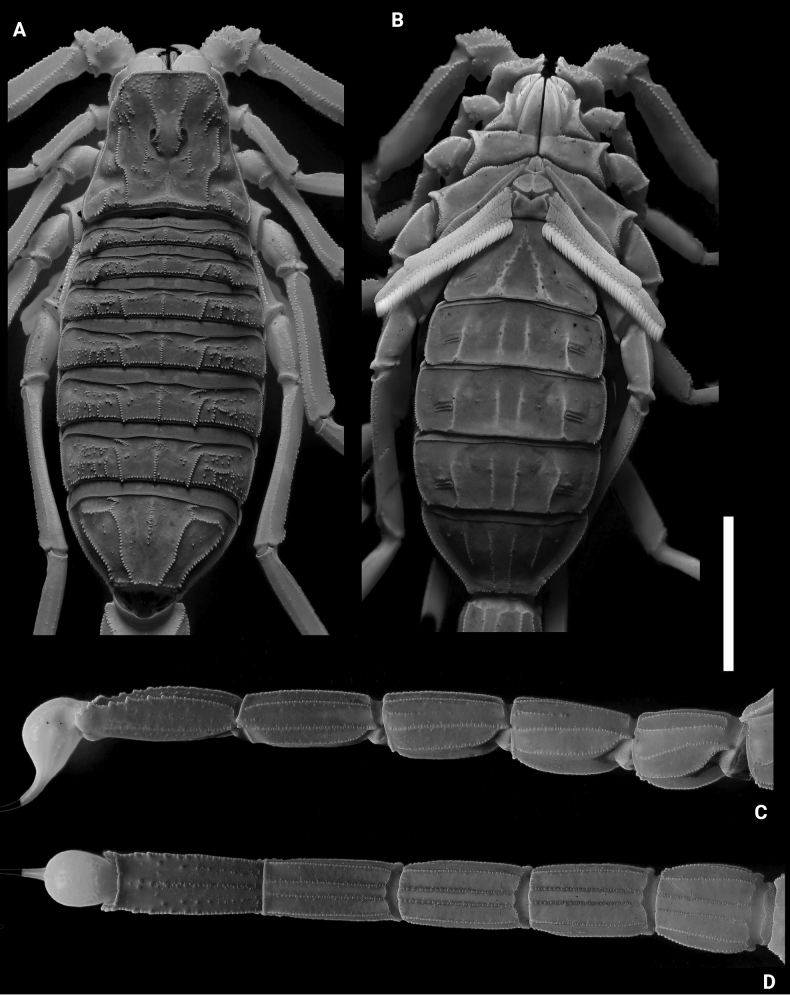
*Leiurushadb* sp. nov., male holotype under UV light **A** carapace and mesosoma **B** sternopectinal area and ventral of mesosoma **C** metasoma and telson, lateral view **D** metasoma and telson, ventral view. Scale bar: 10 mm.

**Figures 3.**
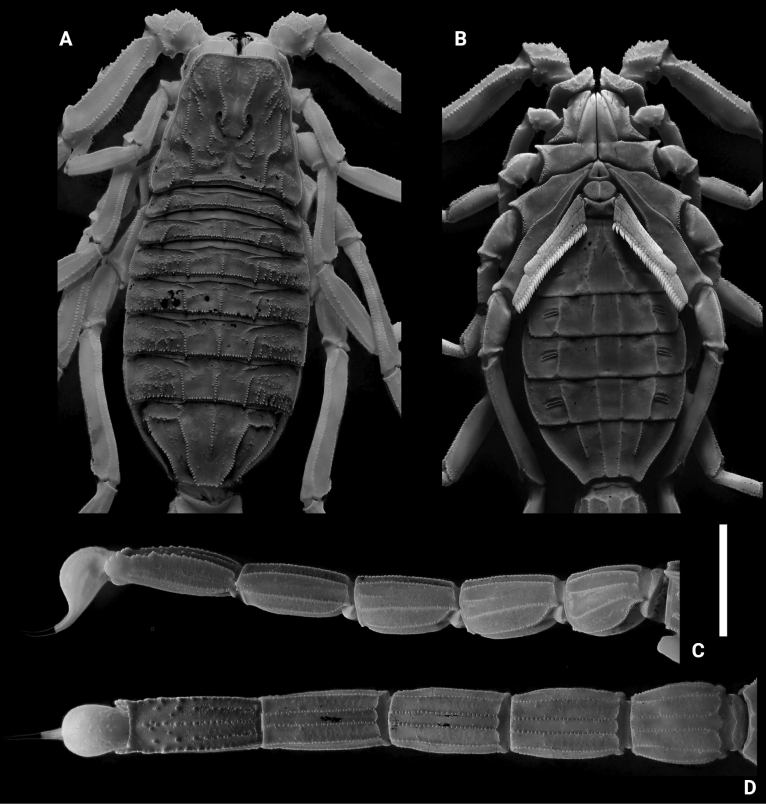
*Leiurushadb* sp. nov., female paratype under UV light **A** carapace and mesosoma **B** sternopectinal area and ventral of mesosoma **C** metasoma and telson, lateral view **D** metasoma and telson, ventral view. Scale bar: 10 mm.

**Figures 4.**
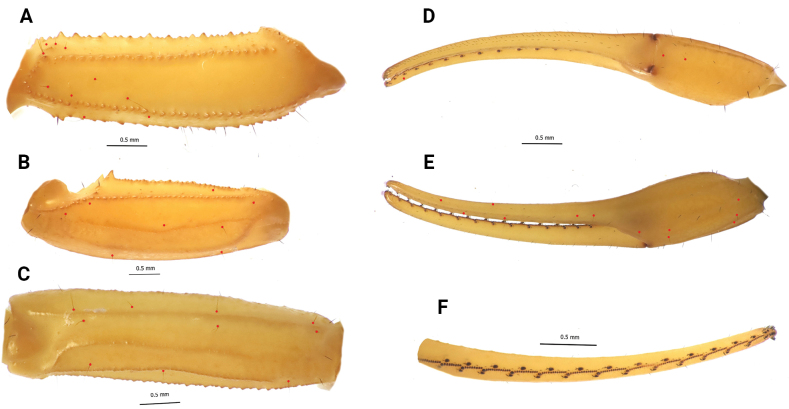
*Leiurushadb* sp. nov., female paratype, pedipalp segments **A** femur, dorsal view **B** patella, dorsal view **C** patella, external view **D** chela, ventral **E** chela, dorso-lateral view **F** chela, movable fingers dentition, trichobothrial pattern indicated by red circles.

**Figures 5.**
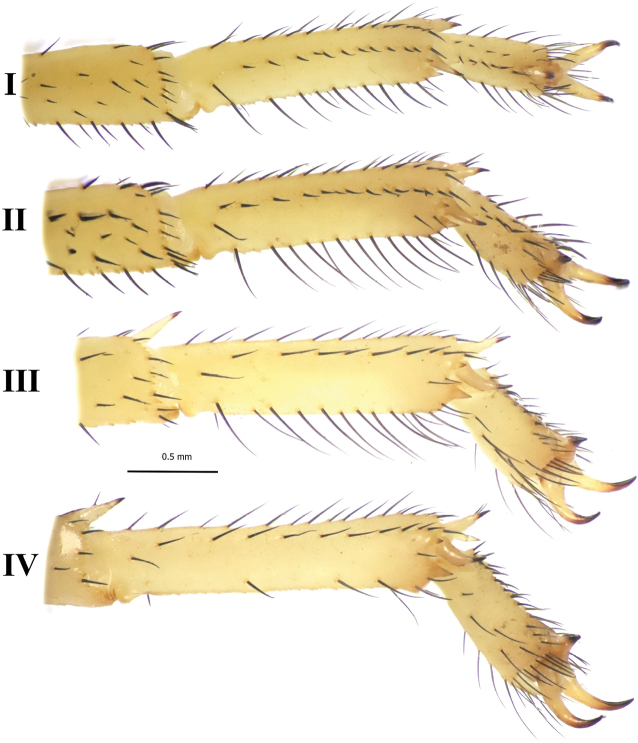
*Leiurushadb* sp. nov., female paratype, right legs I–IV, retrolateral aspect.

**Figures 6.**
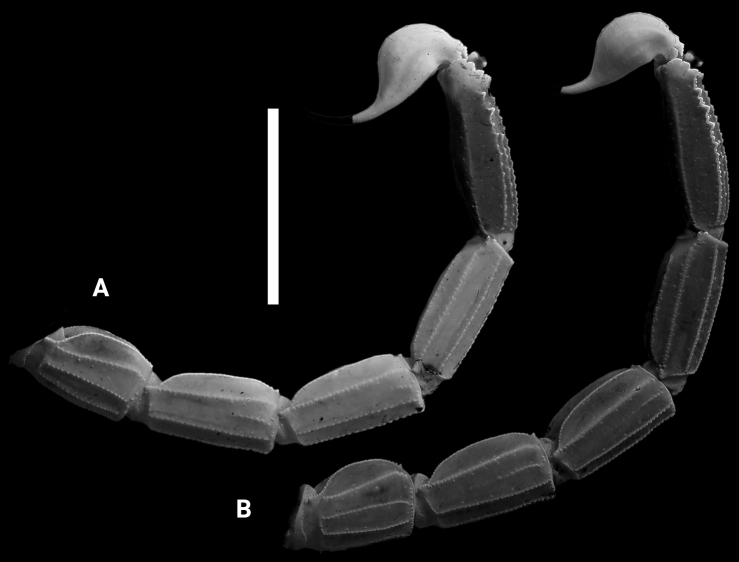
Comparison of metasoma I–V and telson between **A** female *L.arabicus*, from Riyadh, Saudi Arabia, and **B** female paratype *Leiurushadb* sp. nov.

**Figure 7.**
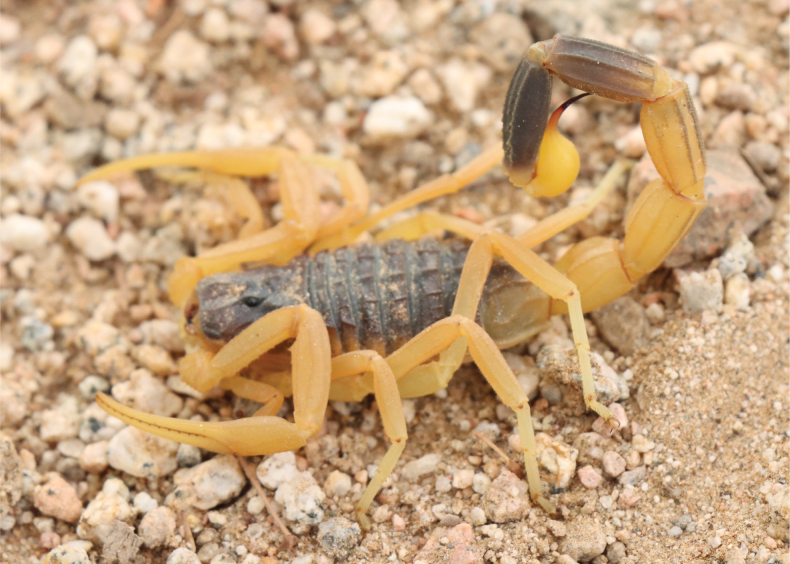
*Leiurushadb* sp. nov., from Wadi Rawdat al-hadb, Riyadh region Saudi Arabia.

Figs 1 , 2 , 3 , 4 , 5 , 6 , 7 , Table 1

#### Material examined.

***Holotype***: ♂ Saudi Arabia, Riyadh: Majami al-Hadb Protected Area, Wadi Rawdat al-hadb (NCWM/Sco-2023:1020), 23.V.2023, 21.605867°N, 43.766051°E, 1045 m a.s.l., Badry A. leg. (NCWM/Sco-2023: 1020). ***Paratypes***: 1♀, Wadi Rawdat al-hadb (NCWM/Sco-2023: 1021), 22.V.2023, 21.650736°N, 43.704530°E, 985 m. a.s.l., Badry A. leg.; 6♀ & 3 juv., Wadi Rawdat al-hadb, 23.V.2023, 21.605867°N, 43.766051°E, 1045 m a.s.l., Badry A. leg. (NCWM/Sco-2023: 1022-29).

#### Comparative material.

*Leiurusarabicus*: Saudi Arabia, Riyadh, 24.253561°N, 46.890831°E, 1♀, Alqahtani AR & Badry A leg. (NCWM/Sco-2023: 1111).

#### Etymology.

The specific name is placed in apposition to the generic name and refers to Majami al-Hadb protected Area, National Center for Wildlife, where the new species was found.

#### Diagnosis.

Medium to large *Leiurus*, 66.5–113.00 mm in length, carapace L 6.7–10.6 mm; base color is yellow or yellow-orange, carapace and tergites with extensive dark pigmentation; ventromedian carinae of metasomas II and III with some vestigial blackish spots over ventral carinae; metasoma IV fuscous except anteriorly; metasoma V heavily blackish; carapace with area between anterior median carinae bearing scattered fine to medium granules, area between posterior median carinae with deep median furrow of carapace moderately flanked by lateral granules by arcs; medial intercarinal surfaces of tergites II and III smooth or lightly shagreened; posterior margin of coxa III smooth or with sparse fine granules; metasoma moderately slender, metasoma II L/W 1.66–1.86, metasoma III L/W 1.81–2.07, metasoma IV L/W 2.02–2.36; ventromedian carinae of metasoma II and III with 23–33 denticles (16/16 carinae); metasoma V with enlarged subtriangular or lobate denticles on ventrolateral carinae; pedipalps slender, patella L/W ♂ 3.62, ♀ 3.08–3.74; leg III patella L/D ♂ 4.10, ♀ 4.16–5.55; pectin teeth ♂ 36–37, ♀ 29–32; pectines long, narrow, pectine L/carapace L ♂ 1.40, ♀ 1.03–1.33, mid-pectine sensillar margin L/metasoma I W ♂ 0.22, ♀ 0.107–0.163; pectin basal piece smooth in females, smooth or slightly shagreened in males; leg III basitarsus with 8–10 retrosuperior setae; pedipalp chela fixed finger with trichobothrium db distal to est; sternite VII with area between median carinae smooth or with sparse fine granulation anteriorly, more heavily in males; sternite carination: males, sternite III with median carinae weak to obsolete, sternites IV and V with weak, finely granulated lateral carinae, obsolete median carinae; females, sternite III with median carinae weak or obsolete, sternites IV and V with lateral carinae moderate, median carinae weak or obsolete.

#### Description

**(based on holotype and paratypes).** Morphometric values presented in Tables 1, 2.

**Table 1.** Morphometric values (in mm) of the female holotype and one male paratype of *Leiurushadb* sp. nov. from Majami al-Hadb Protected Area.

	*Leiurushadb* sp. nov.	*Leiurushadb* sp. nov.
	♂ holotype	♀ paratype
Total length (including telson)	85.5	92.00
Carapace:		
- length	8.5	9.4
- anterior width	6.1	6.4
- posterior width	10.6	11.2
Mesosoma length	24.2	22.4
Metasomal segment I:		
- length	7.2	8.1
- width	5.3	6.4
Metasomal segment II:		
- length	7.9	9.0
- width	4.6	4.3
Metasomal segment III:		
- length	8.1	9.4
- width	4.2	5.2
Metasomal segment IV:		
- length	7.9	9.7
- width	3.8	4.8
Metasomal segment V:		
- length	10.0	11.7
- width	3.6	4.3
- depth	3.5	4.0
Telson length	9.4	9.1
Vesicle:		
- length	5.3	6.1
- width	3.5	4.1
Pedipalp:		
- Femur length	9.7	10.1
- Femur width	2.4	2.9
- Patella length	10.5	11.3
- Patella width	2.9	3.4
- Chela length	18.6	19.9
- Chela width	2.7	3.4
- chela manus ventral		
- length	5.7	6.3
Movable finger:		
- length	13.2	14.0

**Table 2.** Morphometric ratios of the ♂ holotype and ♀ paratype of *Leiurushadb* sp. nov. from Saudi Arabia, including the ranges, mean ± SD, and sample sizes (in parentheses) for length (L) and width (W).

Ratio	*Leiurushadb* sp. nov.	*Leiurushadb* sp. nov.
	♂ holotype (*N* = 1)	♀ paratype (*N* = 7)
Carapace W/ L	1.24	1.14–1.24 1.19 ± 0.038
Pedipalp femur L/W	4.04	3.33–4.16 3.79 ± 0.30
Pedipalp patella L/W = *b*	3.62	3.08–3.743 3.35 ± 0.23
Pedipalp chela L/manus W	6.88	3.18–6.66 5.74 ± 1.16
Pedipalp movable finger L/ manus ventral L	2.31	2.08–2.87 2.28 ± 0.26
Pedipalp movable finger L/ carapace L	2.31	2.08–2.87 2.28 ± 0.26
Pedipalp chela manus W/ carapace L	0.31	0.30–0.66 0.37 ± 0.13
Leg III patella L/D = *c*	4.10	4.15–5.54 4.83 ± 0.57
Pectine L/ carapace L	1.43	1.03–1.32 1.14 ± 0.10
Metasoma I L/W	1.35	1.26–1.39 1.31 ± 0.05
Metasoma II L/W	1.71	1.65 ± 1.85 1.74 ± 0.07
Metasoma III L/W = *a*	1.92	1.80–2.06 1.89 ± 0.10
Metasoma IV L/W	2.07	2.02–2.36 2.20 ± 0.10
Metasoma V L/W	2.77	2.57–2.72 2.24 ± 0.05
Mid-pectine sensillar margin L/ metasoma I W	0.22	0.10–0.16 0.13 ± 0.01
Fs = *a.b.c*	28.66	23.57–37.61 25.73 ± 6.36

***Coloration*.** Base color yellow or yellow-orange, carapace and tergites with extensive dark pigmentation; carapace dark on anterior interocular area, carinae, and posterior margin, light around posterior median furrow, lateral flanks outside lateral carinae pale; pretergites I–VI dark posteriorly, maculate anteriorly, III–VI with pair of pale median spots; tergites I–VI fuscous on medial and mediolateral intercarinal surfaces, with pair of pale anterior median spots, lateral flanks pale; tergite VII with slight fuscosity in anterior median area; ventromedian carinae of metasomas II and III with some vestigial blackish spots over ventral carinae; metasoma IV fuscous except anteriorly; metasoma V heavily blackish.

***Prosoma*** (Figs 1–3). Subrectangular, broad, W/L 1.19, with moderately sloped lateral flanks; upper surface with nearly flat posterior and medial plateau, strongly raised ocular tubercle; interocular triangle convex laterally, depressed medially; anterior margin very slightly emarginate, nearly straight, medially microdenticulate, bordered by row of medium sized granules; eight short macrosetae on anterior margin, carapace otherwise devoid of macrosetae; five lateral eyes (3 large, 2 small) on each side; carination: anterior median, superciliary, central lateral, posterior median and posterior lateral carinae moderate to strong, coarsely granular; anterior median carinae not extending to anterior margin of carapace, separated from anterior marginal row of granules; central lateral and posterior median carinae fused into lyre configuration; central median carinae coarsely granular, anterior part nearly straight, angled outward, posterior part outwardly curved; posterior lateral carinae strong, hind end without lateral extension, projecting only slightly past posterior margin of carapace; lateral ocular carinae moderate, with medium, spaced granules; granulation: sparse patches of 14–16 small to large granules on anterolateral corners of interocular triangle, 8–14 small to medium granules in front of lateral ocular carinae; surface between anterior median carinae smooth except for ten small to medium granules dispersed in anterior area; other intercarinal surfaces smooth except for few isolated small to medium granules; posterior median furrow shallow, broad, with few median microgranules, flanked by lateral arcs of small granules; posterior margin of carapace between posterior lateral carinae rimmed by regular series of medium granules.

***Chelicera*.** Dorsal surface of manus smooth, with six short, pale microsetae, four near apical margin, two subapical, each surrounded by granules; dorso-internal carina at base of fixed finger very strong, well granulated, terminating anteriorly with prominent granules projecting over front of manus; single macroseta in middle of dorso-internal carina; dorsal surface of movable finger smooth, with four pale microsetae; fingers with characteristic buthid dentition (Vachon, 1963); movable finger dorsal margin with five teeth: dorsal distal tine, subdistal, median and two basal teeth fused in bicuspid; ventral margin with three teeth: ventral distal tine, median and basal teeth; fixed finger margin with four teeth: distal tine, subdistal, median and basal teeth; ventral aspect of fixed finger with two teeth.

***Pectines*** (Figs 1–3) long; pectinal tooth count 31–32 for female holotype and 36–37 for one male paratype.

***Mesosoma*** (Figs 1–3). Mesosomal pretergites smooth; tergites: tergites I and II with five granular carinae; III and VI with three straight or slightly curved carinae with medium granules; all carinae moderate to strong, terminating distally in a spinoid process that extends slightly beyond the posterior margin of the tergite; median carinae moderate on I, moderate to strong on II–VI; tergite VII with five strong, granular carinae, with lateral pairs of carinae moderate to strong and joined anteriorly by transverse granule rows; fine granulation on anterior median patch and transverse strips on either side; intercarinal surface smooth, with transverse anterior series of small or medium granules; very fine granulation on anterior median patch and in transverse strips on either side; sternites: sternite III with median carinae weak to obsolete; sternites IV and V with strong, finely granulated lateral carinae, weak to vestigial median carinae; sternite VI with strong, coarsely granulated lateral carinae, moderate, finely granulated median carinae; sternite VII with strong, coarsely granulated median and lateral carinae; medial intercarinal surfaces of all sternites smooth or lightly shagreened anteriorly, lateral intercarinal surfaces smooth posteriorly, lightly shagreened anteriorly on sternites IV–VI.

***Metasoma*** (Figs 1–3). Metasomal segments I–III with ten carinae lateral inframedian carinae complete on I; restricted to posterior zone by 1/2 of the length on II and III; IV with eight carinae. Dorsal and dorsolateral carinae moderate, without any enlarged denticles distally; ventromedian carinae moderate on I and IV, moderate anteriorly, strong posteriorly on II and III, with posterior granules taller but shorter; 25–33 granules on ventromedian carinae of metasoma II and III; metasomal segment V with five carinae; metasomal segment V with five carinae; dorsolateral carinae very weak, faintly granulated, ventrolateral carinae strong with rounded dentate granules increasing in size posteriorly, with several large subtriangular, lobate denticles, ventrosubmedian carinae marked by prominent series of medium to large rounded, dentate granules along length of segment, ventromedian carina strong, with medium to large rounded, dentate granules increasing in size posteriorly; anal arch with three slightly spinoid lobes; intercarinal spaces almost smooth, with only a few granules on the ventral surface of segment V. Telson smooth; subaculear tubercle absent; aculeus with a similar length to that of vesicle; weakly curved. Chelicerae fingers exhibit characteristic buthid dentition (Vachon 1963).

***Pedipalps*** (Fig. 4). Femur moderately slender, L/W 3.28; with five carinae; all carinae strong with coarse, closely spaced dentate granules; internal carina strong, with small and large dentate granules spaced well apart; external carina moderate, with well-spaced, coarse, dentate granules; patella with seven carinae; moderately slender, L/W 3.32; all carinae moderately to weakly crenulate; dorso-internal carinae with four or five small spinoid granules; all intercarinal surfaces smooth; chela slender, smooth and without carinae, L/W 5.85, with elongated fingers, movable finger L/ manus ventral L 2.2;. denticle subrows of fixed and movable fingers composed of 12–12 almost linear rows of granules in a large majority of the examined specimens.

***Trichobothriotaxy*** (Fig. 4). Trichobothrial pattern orthobothriotaxic, type A (Vachon 1974); dorsal trichobothria of femur in β (beta) configuration (Vachon 1975). Dorsal trichobothrium of femur *d_4_* slightly distal in relation to the external trichobothrium *e_1_*; chela fixed finger trichobothrium *db* distal to *est*.

***Legs*** (Fig. 5) moderately long, slender, patella III L/D 4.19; inferior carinae strongly denticulate on femurs I–IV and patellas I–III, very weakly denticulate, almost smooth on patella IV; tibiae III and IV with long spurs; retrolateral tarsal spurs simple, non-setose; prolateral tarsal spurs basally bifurcate, bearing 1–3 macrosetae; basitarsi I–III with well-developed bristle-combs.

#### Habitat and ecology.

**Figure 8.**
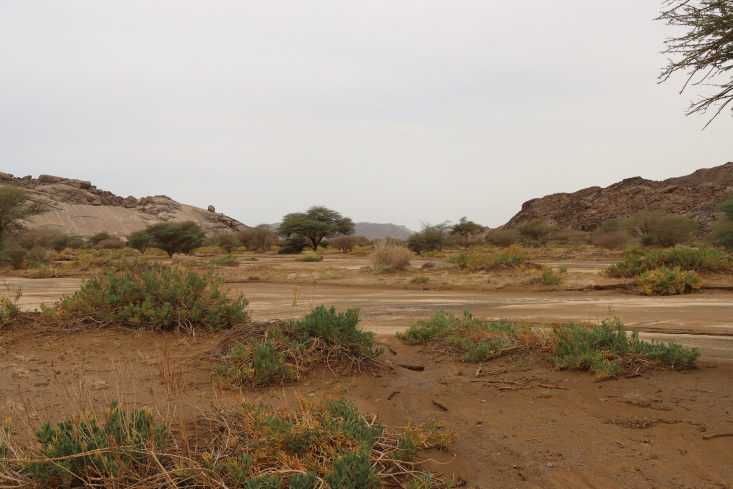
Habitat at the type locality of *Leiurushadb* sp. nov., from Wadi Rawdat al-hadb, Riyadh region Saudi Arabia.

Most collections came from vegetated wadis in Majami al-Hadb Protected Area arid deserts. The species is probably lapidicolous, living under rocks in wadis of both sedimentary and igneous hills and mountains (Fig. 8).

The Mujam’a Al-Hadab protected area is situated in the southwest of the Al-Hamra Mountains, approximately 80 km from the city of Rania in the Riyadh region. Covering 2256 square kilometers, it is dominated by dark volcanic mountains, sandy desert plains, and faded granite domes. These features give rise to wadis such as Wadi Sdiri, Wadi Al-Hamal, and Wadi Al-Farsha. The granite domes in this area range in color from pink to gray and have a smooth texture. Some of these domes rise ~ 400 meters above their surroundings. Desert air circulation has created cavities in the dome facades, along with small caves that fill with water during rainfall and persist for months after the rain stops. In addition to other scorpion species, *Androctonuscrassicauda* (Olivier, 1807) and *Compsobuthusmanzonii* (Borelli, 1915), can also be found in these mountains.

##### ﻿Relationships

*Leiurushadb* sp. nov. differs from *Leiurusquinquestriatus* and other Saudi Arabian species in the following characters:

1. It differs from *L.arabicus*, *L.brachycentrus*, *L.haenggii* and *L.quinquestriatus* that color pattern of the metasoma IV fuscous except anteriorly and metasoma V heavily blackish.
2. It differs from *L.quinquestriatus* in that posterior medial area of carapace with shallow to moderately deep median furrow, versus flat, flanked by lateral granule arcs.
3. It differs from *L.heberti* in metasoma III ventromedian carinae with < 35 denticles.
4. It differs from *L. brachycentrus and L. jordanensis* that medial intercarinal surfaces of tergites II and III between granule clusters smooth to sparsely shagreened or granulated. Also, the medial intercarinal surfaces of sternites being smooth or lightly, finely shagreened versus heavily or densely, finely shagreened.
5. It differs from *L.haenggii* by having more slender leg, pedipalp, and metasomal segments. Biometric separation of adult females of the two species was obtained from the product of three morphometric ratios quantifying slenderness of pedipalp, leg, and metasomal segments: Fs = (pedipalp patella L/W) × (leg III patella L/D) × (metasoma III L/W) > 23 versus (13.95–20.29 for *L.haenggii*) (see Tables 1, 2).
6. It differs from *L.arabicus* by having smooth or weakly granulated median carinae on sternites III–V of females versus weak to moderate; lateral inframedian carinae represented in its posterior zone by 1/2 of the length on II and III versus 0.28 of II, posterior 0.23 on III. Metasoma V with enlarged subtriangular or lobate denticles on ventrolateral carinae (Fig. 6) versus enlarged, triangular or subtriangular.

##### ﻿Genetic analysis

The 16S rRNA data set analysis revealed that of 307 aligned nucleotides, 100 (32.57%) bases were constant, 199 (64.82%) bases were variable, and 80 (26.05%) were parsimony informative. The data set contained 119 polymorphic segregating sites within the 307 bp. The sequence divergences among *Leiurus* lineages ranged from 0.00 to 0.18, with an average of 0.15 (Table 3).

**Table 3.** The uncorrected p-distance of the sequence divergence of 16S mtDNA sequences between *Leiurus* samples was included in this study (standard error shown above the diagonal).

Species	1	2	3	4	5	6	7	8	9	10	11	12	13	14	15	16	17	18
**1. *L.hadb* sp. nov. 1**		0.00	0.00	0.00	0.00	0.02	0.02	0.01	0.01	0.02	0.02	0.02	0.02	0.02	0.02	0.02	0.02	0.03
**2. *L.hadb* sp. nov. 2**	0.00		0.00	0.00	0.00	0.02	0.02	0.01	0.01	0.02	0.02	0.02	0.02	0.02	0.02	0.02	0.02	0.03
**3. *L.hadb* sp. nov. 3**	0.00	0.00		0.00	0.00	0.02	0.02	0.01	0.01	0.02	0.02	0.02	0.02	0.02	0.02	0.02	0.02	0.03
**4. *L.hadb* sp. nov. 4**	0.00	0.00	0.00		0.00	0.02	0.02	0.01	0.01	0.02	0.02	0.02	0.02	0.02	0.02	0.02	0.02	0.03
**5. *L.hadb* sp. nov. 5**	0.00	0.00	0.00	0.00		0.02	0.02	0.01	0.01	0.02	0.02	0.02	0.02	0.02	0.02	0.02	0.02	0.03
**6. La1 *L.arabicus* KSA**	0.11	0.11	0.11	0.11	0.11		0.00	0.02	0.02	0.02	0.02	0.02	0.02	0.02	0.02	0.02	0.02	0.03
**7. La2 *L.arabicus* KSA**	0.11	0.11	0.11	0.11	0.11	0.00		0.02	0.02	0.02	0.02	0.02	0.02	0.02	0.02	0.02	0.02	0.03
**8. Lh1 *L.haenggii* KSA**	0.06	0.06	0.06	0.06	0.06	0.11	0.11		0.00	0.02	0.02	0.02	0.02	0.02	0.02	0.02	0.02	0.03
**9. Lh2 *L.haenggii* KSA**	0.06	0.06	0.06	0.06	0.06	0.11	0.11	0.00		0.02	0.02	0.02	0.02	0.02	0.02	0.02	0.02	0.03
**10. MT111845 *L.quinquestriatus* Egypt**	0.17	0.17	0.17	0.17	0.17	0.15	0.14	0.19	0.19		0.01	0.00	0.01	0.02	0.02	0.02	0.02	0.03
**11. MT111856 *L.quinquestriatus* Egypt**	0.16	0.16	0.16	0.16	0.16	0.16	0.16	0.18	0.18	0.02		0.01	0.00	0.02	0.02	0.02	0.02	0.03
**12. MT111862 *L.quinquestriatus* Egypt**	0.17	0.17	0.17	0.17	0.17	0.15	0.15	0.19	0.19	0.00	0.03		0.01	0.02	0.02	0.02	0.02	0.03
**13. MT111864 *L.quinquestriatus* Egypt**	0.17	0.16	0.17	0.16	0.16	0.16	0.15	0.18	0.18	0.02	0.00	0.02		0.02	0.02	0.02	0.02	0.03
**14. MT111865 *L.quinquestriatus* S. Saini Egypt**	0.18	0.17	0.18	0.17	0.17	0.14	0.15	0.18	0.18	0.10	0.09	0.11	0.09		0.00	0.02	0.02	0.03
**15. MT111866 *L.quinquestriatus* S. Saini Egypt**	0.18	0.17	0.18	0.17	0.17	0.14	0.15	0.18	0.18	0.10	0.09	0.11	0.09	0.00		0.02	0.02	0.03
**16. AY226174.2 *L.macrocentrus* Oman**	0.11	0.11	0.11	0.11	0.11	0.13	0.14	0.12	0.12	0.18	0.18	0.17	0.18	0.18	0.18		0.02	0.03
**17. KU318423.1 *L.abdullahbayrami* Turkey**	0.10	0.10	0.10	0.10	0.10	0.14	0.15	0.10	0.10	0.15	0.15	0.15	0.15	0.14	0.14	0.14		0.03
**18. AJ277598.1 *Androctonuscrassicauda* (Outgroup)**	0.57	0.57	0.57	0.57	0.57	0.55	0.54	0.57	0.57	0.52	0.53	0.52	0.52	0.52	0.52	0.56	0.57	

##### ﻿Phylogenetic analyses

**Figure 9.**
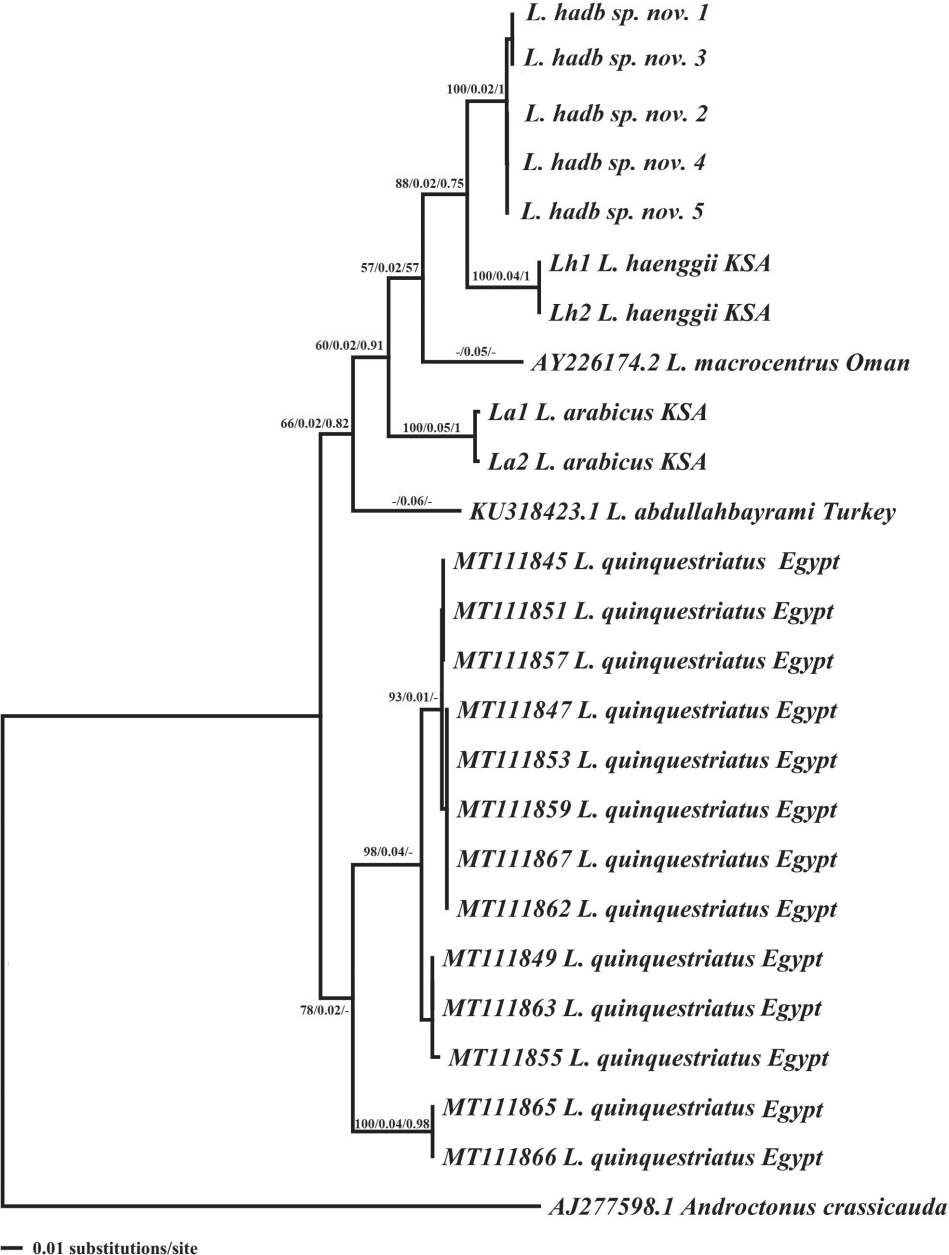
Neighbor-joining (NJ) phylogenetic tree of *Leiurus* species based on 16S rRNA sequences. Numbers above and below branches indicate maximum-parsimony bootstrap values/ NJ distance values /Bayesian posterior probabilities.

The phylogenetic analyses resulted in an identical topology between the maximum-parsimony and the neighbor-joining tree (Fig. 9). However, the general topology of the maximum-parsimony tree was slightly different from those obtained by BI analyses. All phylogenetic analyses (Fig. 9) showed two major clades represented by all *Leiurus* taxa. The first clade includes all Arabian and Middle Eastern forms, which is further split into two subclades: one includes *L.abdullahbayrami* from Turkey and those of the Arabian part (Saudi Arabia and Oman), and the other includes *L.arabicus* from Riyadh as a basal clade to the sequences *L.hadb* sp. nov., *L.haenggii* from southwestern Saudi Arabia, and *L.macroctenus* from Oman, which grouped together as a sister group. The second clade includes all Egyptian forms.

##### ﻿Genetic distance

Table 3 shows the genetic distances from the entire data set, with the lowest divergence (6%) observed between the Arabian species *L.hadb* sp. nov., and *L.haenggii*, and the highest divergence observed between *L.hadb* sp. nov. and other Saudi Arabian *Leiurus* species, ranging from 10% to 12%.

### ﻿Key to species of the genus *Leiurus* from Saudi Arabia

**Table:** 

1	Medial intercarinal surfaces of tergites II–III smooth or sparsely, lightly shagreened	**2**
–	Medial intercarinal surfaces of tergites II–III heavily or densely, finely shagreened	**4**
2	Females with pedipalp patella L/W < 3.20, Fs < 23	***L* . *haenggii***
–	Females with pedipalp patella L/W > 3.20, Fs > 23	**3**
3	Metasomal segment IV fuscous except anteriorly; Smooth or weakly granulated median carinae on sternites III–V of females; lateral inframedian carinae represented in its posterior zone by 1/2 of the length on II and III; metasoma V with enlarged subtriangular or lobate denticles on ventrolateral carinae	***L.hadb* sp. nov.**
–	Metasomal segment IV yellow; Moderate to strongly granulated median carinae on sternites III–V of females; median lateral carinae restricted to posterior 0.28 of II, posterior 0.23 of III; metasoma V with enlarged, triangular, or subtriangular denticles on ventrolateral carinae	***L* . *arabicus***
4	Metasoma III ventromedian carinae with > 30 denticles; metasoma I–IV uniformly fuscous	***L.jordanensis***
–	Metasoma III ventromedian carinae with < 30 denticles; metasoma I–IV yellow to light	***L.brachycentrus***

## ﻿Discussion

Our results indicate morphological differences between *L.hadb* sp. nov., *L.arabicus*, and *L.haenggii*. *L.hadb* is closely related to *L.arabicus*, as they share slender leg, pedipalp, and metasomal segments (Lowe et al. 2014). However, *L.hadb* differs from *L.arabicus* in several diagnostic characters, includes; *L.hadb* has smooth or weakly granulated median carinae on sternites III–V of females, whereas *L.arabicus* has weak to moderate carinae. Additionally, the lateral inframedian carinae of *L.hadb* are represented in the posterior zone by 1/2 of the length on II and III, while L.arabicus has 0.28 of II and posterior 0.23 on III. Lastly, metasoma V of *L.hadb* has enlarged subtriangular or lobate denticles on ventrolateral carinae (Fig. 6), whereas *L.arabicus* has enlarged triangular or subtriangular denticles.

Also, we conducted a molecular phylogenetic analysis using the mRNA 16S mitochondrial gene. Our analysis revealed a genetic divergence between *L.hadb* sp. nov. and samples of *L.arabicus* and *L.haenggii* from Saudi Arabia (*p*-distance = 0.06–0.012; Table 3). Also, the phylogenetic trees were topologically consistent (Fig. 9), and we found that *L.hadb* sp. nov. was most closely related to the *L.haenggii* sequence from southwestern Saudi Arabia. However, *L.haenggii* can be distinguished from *L.hadb* sp. nov. and *L.arabicus* by having more robust leg, pedipalp, and metasomal segments (Lowe et al. 2014). Several authors have used the 16S mitochondrial gene to identify cryptic species of *Euscorpius* Thorell, 1876 and *Centruroides* Marx, 1890 (Fet et al. 2003; Quijano-Ravell and Ponce-Saavedra 2016). It is suggested that the genetic divergence between *L.hadb* sp. nov. and the other *Leiurus* species from Saudi Arabia is likely due to a combination of physical barriers and ecological differences (Alqahtani and Badry 2021). According to (Lowe et al. 2014), *L.arabicus* and *L.haenggii* are closely related parapatric forms that are distributed in adjacent ecological regions and habitats of the Arabian Peninsula. *Leiurusarabicus* is distributed belong the alluvial desert plains of the central Najd plateau and eastern plains extending to the Gulf coast, while *L.haenggii* is found in the rocky mountains along the Red Sea coast and Hadramout. Lowe et al. (2014) referred to the possibility of hybridization in transition zones between *L.arabicus* and *L.haenggii* which raises interesting questions about the evolutionary history of these species and the factors that may have contributed to their differentiation. The divergence could be explained by a vicariant event caused by progressive aridification during the Late Pleistocene and early Holocene, which led to diversification among Arabian species (Alqahtani and Badry 2020a, b; Sarhan et al. 2020). In addition, the genetic isolation of these species is further evident in the divergent evolution of polypeptide toxins in their venoms (Smertenko et al. 2001) as well as different physicochemical profiles of their venom proteins (Nascimento et al. 2006). Also, previous studies have reported that paleoclimatic conditions had a significant impact on the distribution and differentiation of various species such as *Androctonus* and *Buthus* including their diversification (e.g., Sousa et al. 2010, 2012; Alqahtani et al. 2022a, 2022b). It appears that the new species, *L.hadb* sp. nov., may be restricted to this particular area, although further specimens from additional sites are necessary to fully understand the range of both *L.arabicus* and *L.haenggii*.

## Supplementary Material

XML Treatment for Leiurus hadb

## ﻿Additional information

### Author ORCIDs

Abdulaziz R. Alqahtani https://orcid.org/0000-0003-2682-7965

Ahmed Badry https://orcid.org/0000-0002-2610-2019

### Data availability

All of the data that support the findings of this study are available in the main text.
